# Potential Cross Talk between Autism Risk Genes and Neurovascular Molecules: A Pilot Study on Impact of Blood Brain Barrier Integrity

**DOI:** 10.3390/cells11142211

**Published:** 2022-07-15

**Authors:** Rekha Jagadapillai, Xiaolu Qiu, Kshama Ojha, Zhu Li, Ayman El-Baz, Shipu Zou, Evelyne Gozal, Gregory N. Barnes

**Affiliations:** 1 Department of Neurology, Pediatric Research Institute, Louisville, KY 40202, USA; rekha.jagadapillai@louisville.edu (R.J.); xiaolu.q00019@ncu.edu.cn (X.Q.); kshama.ojha@cmh.edu (K.O.); 2 University of Louisville Autism Center, Louisville, KY 40217, USA; 3 Department of Pediatrics, Pediatric Research Institute, University of Louisville School of Medicine, Louisville, KY 40202, USA; 4 Department of Child Health, Jiangxi Provincial Children’s Hospital, Donghu District, Nanchang 330006, China; ndeky00018@ncu.edu.cn; 5 Department of Neurology, Vanderbilt University School of Medicine, Nashville, TN 37232, USA; zhu.li@vumc.org; 6 Department of Bioengineering, University of Louisville Speed School, Louisville, KY 40292, USA; ayman.elbaz@louisville.edu

**Keywords:** genes, vascular signaling, neural circuits, research domain criteria, cognition, and behavior, neurovascular inflammation, blood brain barrier

## Abstract

Autism Spectrum Disorder (ASD) is a common pediatric neurobiological disorder with up to 80% of genetic etiologies. Systems biology approaches may make it possible to test novel therapeutic strategies targeting molecular pathways to alleviate ASD symptoms. A clinical database of autism subjects was queried for individuals with a copy number variation (CNV) on microarray, Vineland, and Parent Concern Questionnaire scores. Pathway analyses of genes from pathogenic CNVs yielded 659 genes whose protein–protein interactions and mRNA expression mapped 121 genes with maximal antenatal expression in 12 brain regions. A Research Domain Criteria (RDoC)-derived neural circuits map revealed significant differences in anxiety, motor, and activities of daily living skills scores between altered CNV genes and normal microarrays subjects, involving Positive Valence (reward), Cognition (IQ), and Social Processes. Vascular signaling was identified as a biological process that may influence these neural circuits. Neuroinflammation, microglial activation, iNOS and 3-nitrotyrosine increase in the brain of Semaphorin 3F- Neuropilin 2 (Sema 3F-NRP2) KO, an ASD mouse model, agree with previous reports in the brain of ASD individuals. Signs of platelet deposition, activation, release of serotonin, and albumin leakage in ASD-relevant brain regions suggest possible blood brain barrier (BBB) deficits. Disruption of neurovascular signaling and BBB with neuroinflammation may mediate causative pathophysiology in some ASD subgroups. Although preliminary, these data demonstrate the potential for developing novel therapeutic strategies based on clinically derived data, genomics, cognitive neuroscience, and basic neuroscience methods.

## 1. Introduction

Autism spectrum disorder (ASD), a neurodevelopmental disorder of combined genetic and environmental etiology, is characterized by impaired behavioral characteristics and synaptic connectivity, with inter-brain regions hypo-connectivity and intra-brain regions hyper-connectivity [1]. The relationship between these synaptic dysfunctions and neuroinflammation during development in autism is unclear [2,3,4]. A central goal of autism research is to identify infants at high risk of ASD and characterize the mechanisms regulating ASD pathogenesis.

The function of neural circuits can be summarized in three highly organized levels: (1) low-level, local networks, or basic sensory or motor processes (visual, motor, somatosensory, auditory networks); (2) secondary high-level, distributed networks, i.e., higher order cognitive and emotional processes (attention, working memory, language, emotions, semantics, social cognition, etc.); and (3) tertiary transient meta-networks, which mediate complex goal-directed and flexible behaviors and cognition. Both functional neuroplasticity and behavioral variability increase with the complexity of the networks mediating a particular brain function [5]. These are reflected by the explosion of findings from autism genetic studies and insights into genetic mechanisms in neural circuits. Identified genetic mechanisms now include monogenic gene disorders (Tuberous sclerosis complex (TSC), Fragile X, PTEN), pathogenic copy number variants (CNV) (i.e., 15q13,3 duplication, etc.), and small deletions/insertions or single nucleotide variants causing protein damaging mutations with loss or gain of function [6,7]. These mechanisms constitute 40–50% of the pathogenic variants that greatly increase the risk of ASD and are testable in the clinic [8]. Although not widely available, whole genome sequencing may lead to genetic findings in another 10–20% of subjects [9]. Genome studies have revealed 44 loci and up to at least 500–1000 genes associated with nonsyndromic cases. Their interactions and exact roles are complicated by genetic heterogeneity, inter-genetic interactions, incomplete penetrance and phenocopies [6,7]. Knowledge of ASD-related genes makes it possible to cross-index autism phenotypes with neurobiological phenotypes, relating genes to cognition and enabling the creation of a systematic and objective taxonomy of neural circuits in clinical disorders [6,7].

Current knowledge does not cross-index ASD-related genes, dysregulation of signaling pathways, brain function, and concomitant behavior. The advent of genetic imaging approaches, which combines genetic findings with brain imaging to parse the pathophysiological mechanisms of ASD, has made it possible to characterize the neural systems which are directly affected by risk gene variants. Data from these studies point to relevant ASD risk genes that are associated with structural and functional alterations in circuits involved in social, cognitive, and motor phenotypes. Despite numerous efforts to assess ASD on the imaging, genomic, and behavioral levels, until now, no system has been able integrate multi-modal data to both diagnose ASD and locate affected individuals on the spectrum.

Central to this concept is the hypothesis that the genes influencing autism lie along distinct signaling pathways. Luo et al. analyzed 3392 and 4792 autism-related mutations from the Simons Simplex Collection (SSC) and Autism Sequencing Consortium (ASC) collections and showed that hundreds of variants or genes repeatedly converge to several canonical pathways that define recurrent and systematic ASD biological pathways [10]. This report reinforces the notion that gene expression pathways may influence the early interactions of synaptic and connectivity functions as the main sculptors of neural circuitry. Techniques and models must allow for the detection of primary neurodevelopmental gene variants and must be sensitive to secondary compensatory processes which may be the causal trigger for ASD symptoms. Such processes may involve vascular signaling located in pathogenic CNVs, which are risk factors for ASD [11]. They may include emerging links to gamma amino butyric acid (GABA) ergic cell biology, which, when impaired during development, contributes to excitatory/inhibitory imbalance, as seen in ASD [12,13,14]. GABA signaling knock out (KO) in mice perivascular endothelial cells results in seizures and ASD behaviors and impacts angiogenesis, neurogenesis, the radial migration of projection neurons, and tangential migration of GABAergic neurons during development [15].

We recently characterized an ASD-linked gene deletion linked to vascular signaling, the neuropilin-2 knockout (NRP2) mouse, as a novel animal model of autism and epilepsy [16]. In addition, we developed a GABAergic cell specific knockout of the NRP2 ligand, semaphorin 3F (Sema 3F), a secreted member of the class 3 semaphorin family initially identified as a neuronal guidance molecule in the developing brain [17,18]. The semaphorin/neuropilin gene family encodes for guidance cues that control processes and cell motility in a wide variety of tissues including the immune and vascular systems, promotes lymphatic vessel growth, and controls soma migration, axon patterning, and GABA/excitatory synaptogenesis in the CNS during development [18,19,20]. Autistic cellular and behavioral phenotypes were noted in both Sema 3F and NRP 2 knockout mice [16,17]. While excitatory cell-specific knockout of Sema 3F does not affect interneuron numbers, GABAergic interneuron specific deletion is associated with decreased interneuron numbers, autistic behavioral deficits, microglial activation, and increased cerebral reactive oxygen species [17]. Thus, cellular localization and downstream signaling of neurovascular components may play a significant role in the phenotypes inducing autism-like behavior seen in NRP2 and Sema 3F KO mutants [16,17,18,21,22,23,24,25].

Among the identified ASD gene mutations, several are expressed in cells responsible for the formation and function of the blood brain barrier (BBB). BBB and intestinal barrier dysfunction was reported in ASD patients, supporting the hypothesis of a gut–brain axis dysfunction that may underlie gastrointestinal disturbances in some ASD patients [26,27,28]. The central questions are: how are gene variants related to the neural circuits which are relevant to behaviors, and how do they implicate specific signaling pathways in the pathophysiology of ASD? Among many possibilities, a linear model of gene variants, of expression in distinct brain regions composing neural circuits during development, and of altering behavior, is one of simplicity which can be tested in an elegant manner with a combination of preclinical and clinical experiments. These experiments were designed as a multidimensional approach to: (1) detect gene variants associated with specific Research Domain Criteria (RDoC)-defined neural circuits relevant to ASD; (2) demonstrate how the expression of these putative vascular signaling gene variants in specific neural circuits influence responses on parental questionnaires in a clinical cohort; and (3) to understand how the impact of vascular signaling on ASD risk factors may alter molecular pathways and Blood Brain Barrier (BBB) function, thereby altering functional synaptic connectivity and the appearance of autism-like behavior. These data also test the hypothesis that neurovascular signaling may influence GABAergic cell development, neural circuit formation, and behavior through interaction with BBB function.

## 2. Material and Methods

### 2.1. IRB Approvals

All studies and access to databases were reviewed and approved by the NIMH and human subject studies institutional review boards of the University of Louisville (IRB# 20.0746) and Vanderbilt University Medical Center (IRB# 090069).

### 2.2. Clinical Derived Database

**Figure 1:**
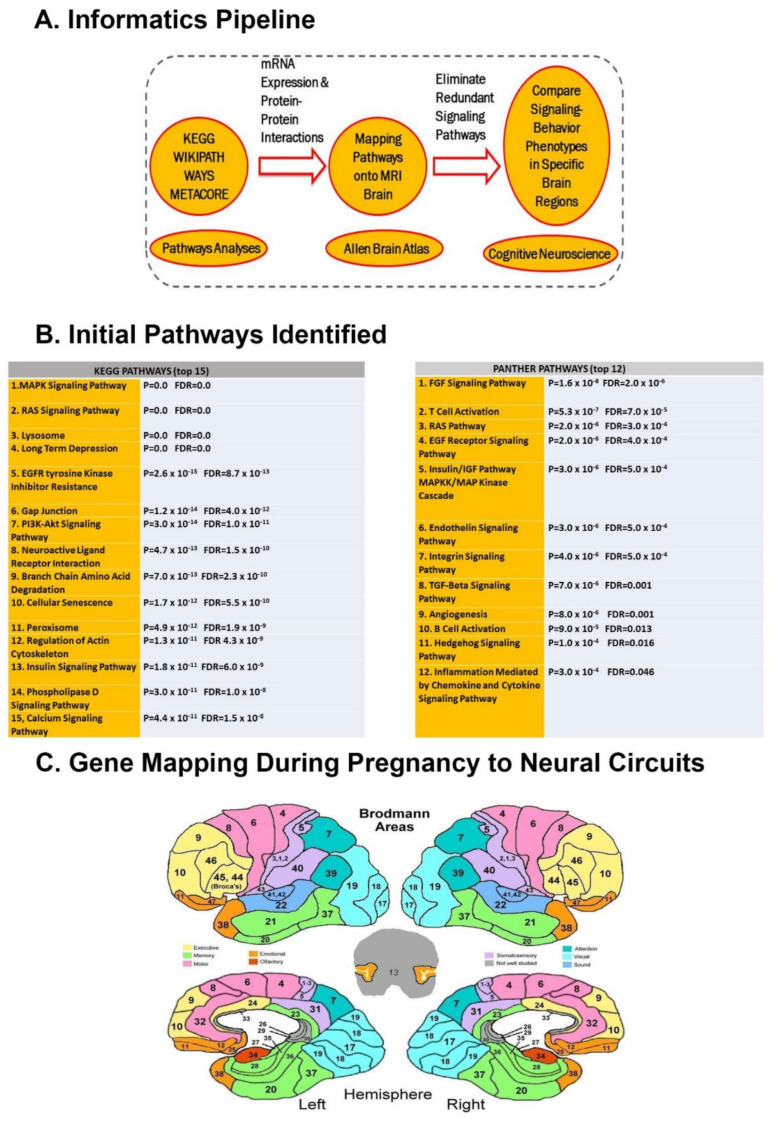
**Informatics Pipeline and Mapping of Genes to Neural Circuits:** (**A**) Schemati representation of the informatic pipeline analysis (**B**) 2631 neurodevelopmental genes, including 594 from pathogenic CNVs of autism subjects, were analyzed via KEGG and Panther pathway analysesProminent signaling pathways which use MAPK, RAS, and tyrosine receptor kinases were common to both analyses. (**C**) After protein–protein interactions were evaluated via String and mRNA Co-expression analyses, 121 genes were mapped for their antenatal/postnatal expression in Research Domain Criteria (RDoC)-defined neural circuits using Brodmann areas.

The electronic medical records of the University of Louisville and Vanderbilt University Medical Center were analyzed for inclusion of pediatric patients who had had a primary diagnosis of autism or both epilepsy and autism, i.e., not secondary to an existing disorder or syndrome. Inclusion criteria, average age, and gender are detailed in the supplemental methods file. Subjects with CNVs consisted of 90 patients (44 with autism alone and 46 with autism and epilepsy), as listed in the supplementary data (Table S1). Thereafter, the genes present in the affected chromosomal loci of the subjects were located based on Human Genome Build 37, which amounted to a total of 594 genes. The analyses pipeline outlined in Figure 1 was used to assure biologically relevant interactions to ASD by requiring multiple levels of evidence in pathway participation, protein–protein interactions, mRNA co-expression, and finally, prenatal peak expression in a given brain region as denoted by the Allen Brain Atlas.

### 2.3. Gene Set Enrichment Analysis

As outlined in Figure 1, 2037 neurodevelopmental genes [29] which have links to neurological and developmental abnormalities were included in the dataset. Along with these neurodevelopmental genes, the 594 genes found in the CNVs from clinical subjects were included as the initial dataset (Total = 2631 genes). The Web based Gene Set Analysis Toolkit (WebGestalt) [30] was used to perform a series of gene set enrichment analyses of the initial dataset of 2631 genes. Gene set enrichment analysis was chosen as a method of interest with the gene symbol being used as the gene identifier. The pathways enrichment analyses were performed using the Kyoto Encyclopedia of Genes and Genomes (KEGG) database. The Homo sapiens genome was used as a reference set in all the above-mentioned analyses. The WebGestalt uses a hypergeometric test for enrichment evaluation analyses and a significance level of 0.001 was chosen for all analyses.

The WebGestalt is a free, web-based integrated data mining system that aids in viewing genes and gene sets in various biological contexts with statistical relevance. It helps to analyze genes and gene sets across various channels that could lead to the identification of interactions or differences and their phenotypic presentations, and also provides evidence to support further research [31]. The KEGG and Panther (Protein ANalysis THrough Evolutionary Relationships) analyses focus on deriving pathways from genetic data, which prove instrumental in determining the presence and extent of pathway–pathway interactions, and aid in establishing a common genetic ground between disorders.

### 2.4. Protein–Protein Interactions

The genes derived from the enrichment analysis using WebGestalt were further evaluated for their protein–protein interactions using Search Tool for the Retrieval of Interacting Genes/Proteins (STRING) [32], as detailed in the supplemental methods file.

### 2.5. mRNA Co-Expression

The set of genes derived from the protein–protein interactions were also analyzed for their mRNA co-expression in the quantile of the Brain Development-Normal Gene Expression (Yale/Sestan) dataset group, using GeneNetwork.org (accessed on 1 January 2019) [33]. The Graph tool was used to generate a correlation graph from the selected traits with a Pearson’s correlation of greater than 0.7 or less than −0.7. GeneNetwork.org (accessed on 1 January 2019) is a free online analytic tool and data reservoir that aids in the integration of largely varied sets of genetic and phenotypic data. It helps to provide an integrated view of genetic and phenotypic data sets [33].

### 2.6. Prenatal Brain Gene Expression Mapping

The set of genes derived from the mRNA co-expression analysis were mapped as per their highest expression during the antenatal period of development of the human brain. This mapping was done using Brainspan.org (accessed on 1 January 2019), which is a component of the Allen Brain Map. Brain Span: Atlas of the Developing Human Brain is a foundational resource for studying the transcriptional mechanisms involved in human brain development. This resource was used to obtain the maximal expression of target genes during specific developmental epochs (first, second, third trimester, postnatal) and to identify which brain region contains the maximal expression of a gene’s mRNA.

### 2.7. Mapping Brain Gene Expression to RDoC Neural Circuits

By integrating these genetic data, the maximum mRNA expression of the gene during pregnancy and post-pregnancy and the brain regions during that period were identified. Brain regions associated with each neural circuit were mapped through NIMH’s RDoC-matrix (https://www.nimh.nih.gov/research/research-funded-by-nimh/rdoc/constructs/rdoc-matrix.shtml (accessed on 1 January 2019)), matched to brain regions with the maximum mRNA gene expression and divided as detailed in the supplemental methods file.

For a given question answered by parents, the scores of ASD subjects with specific genetic variation in a given neural circuit were compared with those with no genetic variation (i.e., ASD subjects with normal microarray: average age 11.2 years) on different behavioral scales. ASD subjects with specific genetic variations were grouped together within the genetic variation group (Group 1) by: (1) specific same gene variation; and (2) maximal mRNA expression in a given brain region contained in the RDoC neural circuits. For each parental concerns questionnaire (PCQ) question or Vineland Scales subscore (Version 2, Comprehensive Interview Form), all RDoC neural circuits in subjects with genetic variants were compared to the scores of ASD subjects with normal microarrays (Group 2).

### 2.8. Statistical Analysis for Neural Circuit Comparisons

All data were collated using SPSS software11 (IBM SPSS Statistics for Windows, Version 24.0. IBM Corp., Armonk, NY, USA) and plots were generated using GraphPad Prism version 8 (GraphPad software, San Diego, CA, USA), as described in the supplemental methods file.

### 2.9. Mouse Experiments

Animal experiments were performed under protocols approved by the University of Louisville Institutional Animal Care and Use Committee (IACUC# 195010).

#### 2.9.1. Mouse Lines

Semaphorin 3F knockout mice (CRE+FF) (DLX5/6^Cre^, deletion in GABAergic Neurons) and control mice (Cre−FF, Cre+WT, Cre−WT) lacking either Cre expression or Flox sites flanking the Sema 3F gene, or both, therefore expressing normal levels of Semaphorin 3F, were described in our previous publication (Li et al., 2019), and were used in the current study. Three to five animals (3 to 6 months old) per group were used for immunofluorescence and immunohistochemical analysis as previously described [17]. Brain of DLX5/6^Cre^ knockouts and of EMX 1^Cre^ heterozygotes (deletion in excitatory neurons), sacrificed at weaning, were weighed and compared to their respective controls.

#### 2.9.2. EEG Recording

Mice were instrumented for 1 week. Then, Cortical EEG Power of 1 min segments/hour for 12 h overnight were measured using Nihon Kohden software spectral analyses features QP-112AK Version 10-03 (Irvine, CA, USA). The value per mouse was the average of 12 measures. N = 5; Differences between means were considered as significant at *p* ≤ 0.05.

#### 2.9.3. Immunofluorescence

Immunofluorescent staining was performed as previously described and detailed in the supplemental methods file [17]. Staining intensity per tissue unit area (μm^2^), as well as co-localization in high magnification images, were quantified and averaged in three regions of interest (ROIs) per slide with three slices per animal using image analysis software (Image-Pro Plus). Data were expressed as intensity per unit area ± SD.

#### 2.9.4. Immunohistochemistry

Immunohistochemistry staining was performed as previously described and detailed in the supplemental methods file [17]. Proteins were detected on color development using 3,3′-diaminobenzidine as substrate or VIP (Vector Laboratories). The images were captured using a Nikon microscope.

#### 2.9.5. Statistical Analysis

GraphPad Prism 6 software (GraphPad Software, San Diego, CA, USA) was used for the statistical analyses. Repeated measures two-way ANOVAs, followed by post-hoc Tukey’s Multiple Comparison Test, were used to compare CRE–FF Sema 3F-KO mice with Cre−FF, Cre+WT, and Cre−WT mice, expressing normal levels of Semaphorin 3F. Data are presented as mean ± SD. Differences between means were considered as significant at *p* ≤ 0.05.

## 3. Results

### 3.1. Analyses of Human Genetic Risk Variants in Children with ASD

Multiple previous pathway analyses of ASD risk genes implied that immune and microglia signaling is important in ASD pathogenesis. These also suggested that neurovascular signaling is important, either causing or perhaps modifying ASD risk [34]. Weighted gene set enrichment analysis on the dataset of 2631 genes using the KEGG pathway database resulted in a network of 61 pathways at a significance level of 0.001. These pathways comprised a group of 659 distinct genes. The most prominent pathways featured in the subject database with respect to the largest number of genes were the mitogen-activated protein kinase (MAPK) and Rat sarcoma virus (RAS) signaling pathways, lysosome, long-term depression, GAP junction, and PI3K-Akt signaling pathway (Figure 1B).

**Figure 2:**
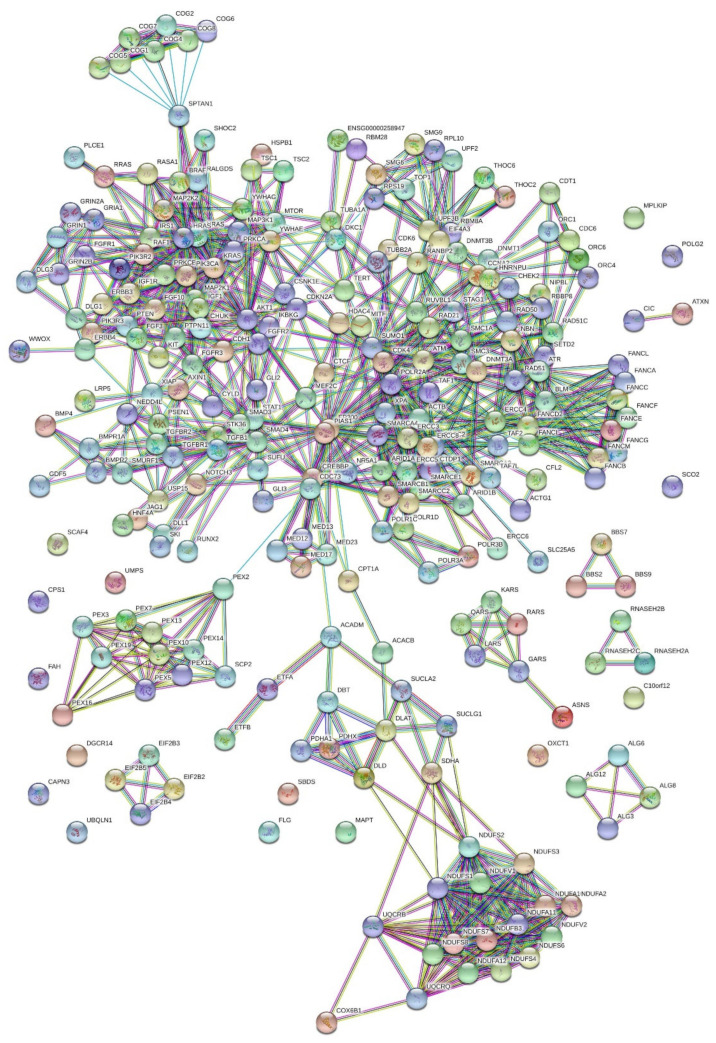
**String Analysis of 659 genes at *p* = 0.001**: 659 genes were evaluated for their protein–protein interactions (at a confidence interval of 0.9), resulting in a subset of 273 genes which were further analyzed for their mRNA co-expression. The ensuing 121 genes (Pearson’s of 0.7). were mapped for their antenatal/postnatal expression in Research Domain Criteria (RDoC)-defined neural circuits using Brodmann areas.

A Weighted Panther Pathway enrichment analysis for protein–protein interactions (PPI) (Figure 2) on the dataset of 659 distinct genes resulted in a network of 12 signaling pathways at a significance FDR < 0.05. The most prominent pathways featured in the subject database with respect to the largest number of genes were the fibroblast growth factor (FGF), RAS, T and B cell activation, endothelin, integrin, transforming growth factor (TGF)-beta, angiogenesis, & Hedgehog signaling pathways (Figure 1B).

After the gene set enrichment analysis, we evaluated the 659 genes for their protein–protein interactions (at a confidence interval of 0.9), providing us with a subset of 273 genes. These were further analyzed for their mRNA co-expression, resulting in a subset of 121 genes (Pearson’s of 0.7) which were then mapped according to maximal expression per antenatal age in the developing human brain (Figure 1C).

For a given question answered by parents, the scores of those ASD subjects (Group 2) with specific genetic variations in a given neural circuit were compared with those of ASD subjects without the specific genetic variations (Group 1). Each of the five specific RDoC neural circuits, i.e., (1) Negative Valence Systems, (2) Positive Valence Systems, (3) Cognitive Systems, (4) Social Processes, and (5) Sensorimotor systems (motor actions, agency and ownership, habit-sensorimotor, innate motor patterns), were probed. Since subject numbers were small (Table 1), those whose gene variants were expressed in the same or related brain regions within an RDoC neural circuits (usually during the same developmental epoch) constituted the genetic variation group (i.e., Group 2).

**Figure 3:**
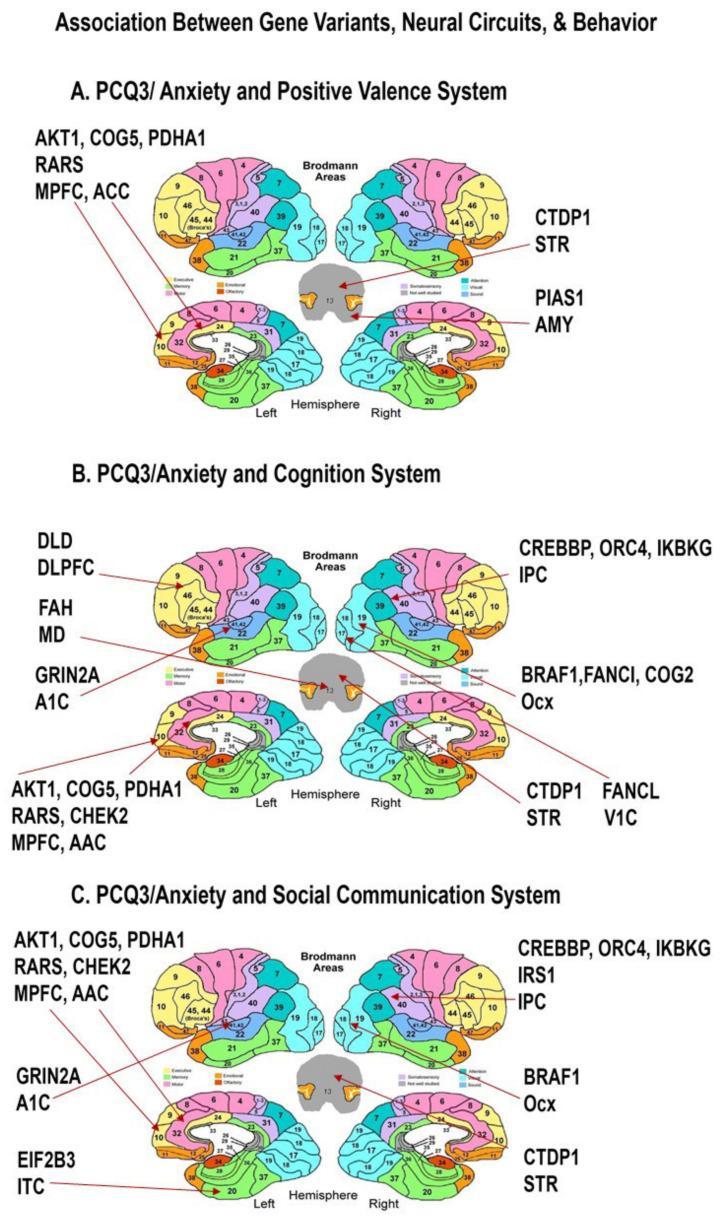
**Neural Mapping of the Genes Associated with Parental Questionnaire Anxiety Scores**. Map of the peak antenatal expression of genes in RDoC neural-derived circuits (as defined by NIMH) and elevated reported levels of PCQ3 anxiety scores. As expected, significantly associated genes are expressed in the Positive Valence (Reward), Cognition, and Social Communication circuits (Table 2). Furthermore, the expression was restricted to a set of Brodmann areas mediating known ASD relevant circuits involving cellular metabolism, neurotrophic, and neuroinflammatory pathways. ACC Anterior Cingulate Cortex, Amy Amygdala, STR striatum, OCx occipital cortex, DLPFC dorsolateral prefrontal cortex, MD mediodorsal nucleus of thalamus, A1C primary auditory cortex, IPC inferior parietal cortex, STR striatum, V1C primary visual cortex (area V1/17), ITC inferolateral temporal cortex (area 20), VLPFC ventrolateral prefrontal cortex.

**Figure 4:**
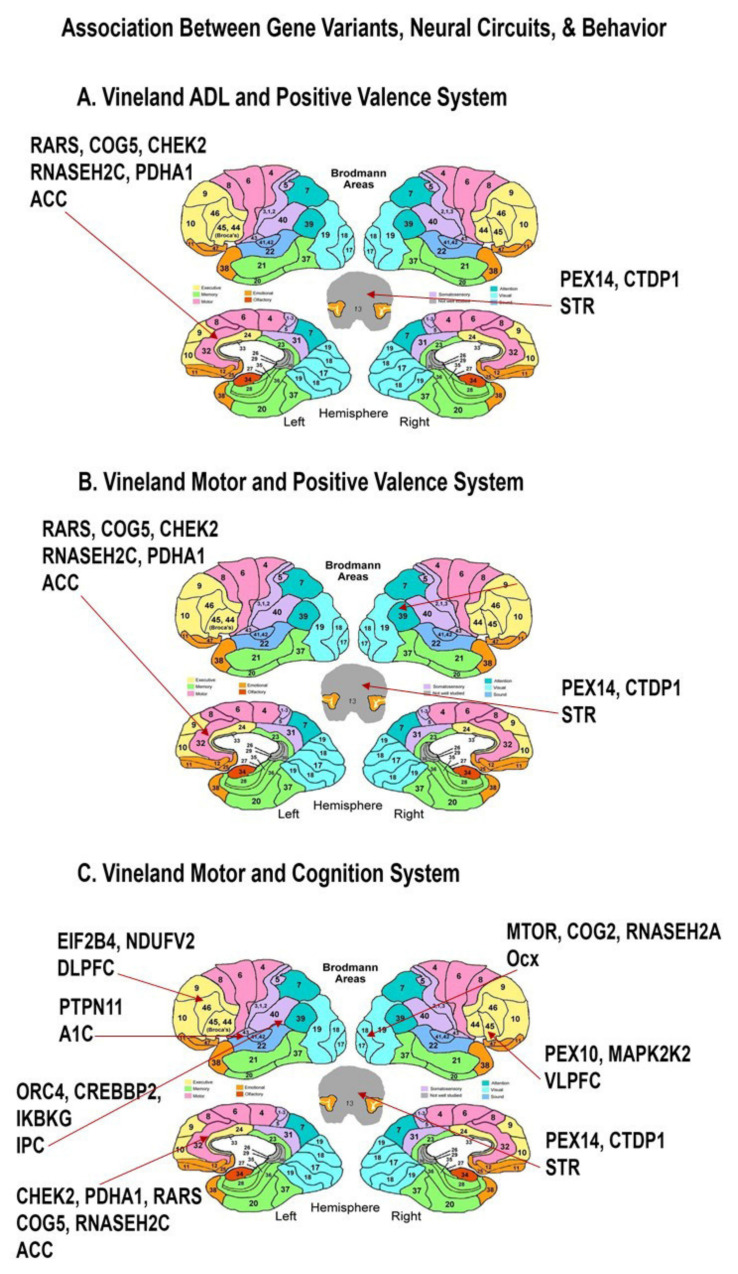
**Neural Mapping of Genes Associated with Elevated Vineland Scores**. Map of the peak antenatal expression of genes in RDoC neural derived circuits (as defined by NIMH) and elevated reported levels of Vineland ADL and Motor scores. As expected, significantly associated genes are expressed in the Positive Valence (Reward), and Cognition neural circuits (Table 2). Similar to the anxiety associated genes, the expression of ADL and motor associated genes was restricted to a set of Brodmann areas mediating known ASD-relevant circuits involving cellular metabolism, neurotrophic, and neuroinflammatory pathways. ACC Anterior Cingulate Cortex, Amy Amygdala, STR striatum, OCx occipital cortex, DLPFC dorsolateral prefrontal cortex, MD mediodorsal nucleus of thalamus, A1C primary auditory cortex, IPC inferior parietal cortex, STR striatum, V1C primary visual cortex (area V1/17), ITC inferolateral temporal cortex (area 20), VLPFC ventrolateral prefrontal cortex.

Panther pathways analyses identified 30+ genes which were expressed in neural circuits that are significantly associated with answers on the PCQ and Vineland (Figure 3 and Figure 4, Table 2 and Table 3). Signaling pathways included: (1) Hypoxia response via hypoxia-inducible factor 1 (HIF-1) activation (*p* = 0.0002); (2) Interleukin signaling (*p* = 0.0003); (3) p53 pathway (*p* = 0.005); (4) CHEK2 signaling (*p* = 0.006); (5) Insulin/IGF (insulin growth factor) pathway-protein kinase B signaling (*p* = 0.01); (6) PI3 kinase signaling (*p* = 0.02); (7) vascular endothelial growth factor (VEGF) signaling pathway; (*p* = 0.03); and (8) RAS pathway (*p* = 0.04) among the whole group comparison. In a whole group comparison, only the parent report of self-stimulatory behavior (PCQ12) on the PCQ demonstrated a significant difference between those with CNVs and those with normal microarrays (Table 2). The Vineland scales and other PCQ questions showed no significant findings between the two groups. However, when we divided the groups into ASD subjects with specific genetic variations among distinct RDoC neural circuits (Table 2 and Table 3), distinct findings were observed with questions related to anxiety on the PCQ and skills on the Vineland related to Activities of Daily Living (ADLs) and Motor function. Anxiety behaviors on the PCQ was significant (Figure 3) in the Positive Valence neural circuits with antenatal expression of two genes, each of which is involved in PI3K/Akt/mTOR-Vit D3 (*p* = 0.00009), Lipid metabolism (*p* = 0.0001), IL-11 (*p* = 0.0003), Amino acid metabolism (*p* = 0.001), androgen receptor (*p* = 0.001), and TGF beta (*p* = 0.003) signaling pathways, within related subcortical (Amy, STR) and limbic system (ACC) regions (Table 3). Cognitive control of anxiety is well known, and cognitive behavioral therapy is well established as a treatment for anxiety [35]. Similarly, anxiety behaviors on the PCQ were significantly associated with worse scores when nine genetic variants with antenatal expression were involved in Estrogen signaling (*p* = 0.000003), DNA IR-damage and cellular response via Ataxia telangiectasia (ATR) (*p* = 0.0001), tumor necrosis factor (TNF)alpha signaling (*p* = 0.0002), B Cell Receptor Pathway (*p* = 0.0003), PI3K/Akt/mTOR-Vit D3 signaling (*p* = 0.0004), and angiogenesis (*p* = 0.0004) in the Cognitive Systems brain regions (Figure 3, Table 3). Social communication is often linked to anxiety [36]. Anxiety PCQ behavioral scores from parents were significantly worse with antenatal expression of 11 gene variants involved with estrogen (*p* = 0.000005), leptin (*p* = 0.0002) and TNF alpha signaling (*p* = 0.0003)) within social communication regions (Figure 3, Table 3).

The Vineland Adaptive Behavior scales have been previously negatively correlated with duplication CNV load in affected ASD children, with the strongest correlation for communication and socialization scores [37]. In this study, the strongest statistical differences were observed for the ADL scale and the motor scale but not the social or communication scales (Table 2). Adaptive function skills are impaired in ASD, and intensive occupational therapy may improve function [38]. Vineland ADL and motor scores from parents were significantly worse when eight gene variants associated with the Positive Valence System and involved with cellular biosynthetic processes were observed but no pathway categories were enriched except for amino acid metabolism (*p* = 0.001) (Figure 4, Table 3). The Vineland motor scale was significantly associated with worse scores in the Cognition System neural circuits when 18 gene variants enriched in Leptin signaling (*p* = 0.000004), IL-6 signaling (*p* = 0.00007), Ataxia Telangiectasia Mutated (ATM) Signaling Network in Development and Disease (*p* = 0.00008), Thymic stromal lymphopoietin (TLSP) signaling (*p* = 0.00009), Brain-derived neurotrophic factor (BDNF) signaling (*p* = 0.0001), interleukin-1 (IL-1) signaling (*p* = 0.0002), and Oncostatin M signaling (*p* = 0.0002) were expressed in the antenatal frontal, auditory, cingulate, striatum, parietal, and occipital cortices (Figure 4, Table 3).

In the Simons Foundation Autism Research Initiative (SFARI) database, three genes (BRAF, GRIN2A, MTOR) are identified as strong autism risk genes. Further, signaling pathways with strong candidate autism genes are the MAP kinase pathway and multiple Interleukin signaling pathways, which were identified in both CNVs (Figure 1) and gene-neural circuit-behavior analyses (Table 2 and Table 3).

### 3.2. Analyses of Neurovascular Signaling and Blood Brain Barrier Integrity in Mouse Models of Neurodevelopment Disorders and Epilepsy

In agreement with numerous neuropathological studies, the data detailed in Figure 1, Figure 2, Figure 3 and Figure 4 and pathway analysis of genes identified in Table 2 and Table 3 demonstrate an association between neurovascular signaling and RDoC Neural circuits/clinical phenotypes whose genetic risk variants are expressed antenatally within defined brain regions. These data suggest that neurovascular signaling influences the RDoC Neural circuits responsible for autism-associated behaviors. Given the role of axonal guidance cues such as semaphorin-neuropilin signaling in the development of these neural circuits and vasculogenesis, we examined neurovascular parameters in the interneuron-specific Sema 3F KO mouse that we previously showed to display ASD-related behavioral features, to further interrogate this hypothesis.

#### 3.2.1. Gross Anatomy and Electrophysiological Assessments

We previously reported the effect of the cell specific deletion of Sema 3F in interneurons and excitatory neurons on electrophysiological and behavioral characteristics in mice [17]. An assessment of brain and body weight at P21 revealed that the DLX5/6^Cre^ CRE+FF mice had a brain weight 10% significantly lower than Cre-FF mice (Table 4). Such brain weight differences did not result in an overall change in the brain/body ratio of interneuron specific (DLX5/5^Cre^) KO and there were no changes in the excitatory neuron specific (EMX1^Cre^) KO (Table 4). Consistent with the increased spikes on cortical EEG, DLX5/5^Cre^ KO mice had a 35% or 58% decrease in the alpha and beta frequency EEG power respectively compared to CRE–FF mice (Table 5). In contrast, CRE+FF mice had no changes in the delta or theta frequency EEG power compared to CRE-FF littermates (Table 5). These data strongly suggest that deletion of Sema 3F in interneurons results in processes during development which impair brain weight and lower EEG power in frequencies normally associated with interneuron regulation of network function.

#### 3.2.2. Immunofluorescence Assessment of Oxidative Stress and Inflammation

**Figure 5:**
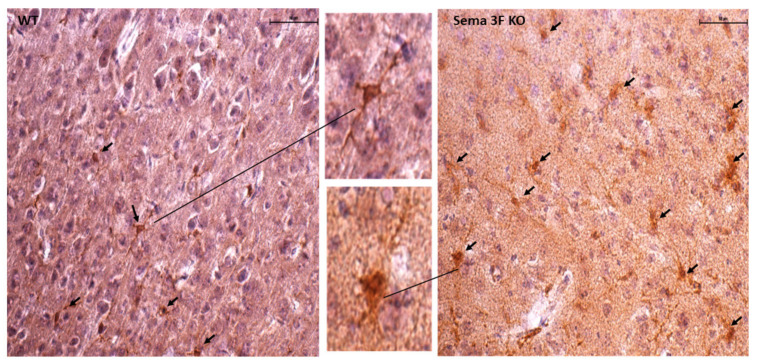
**Microglial Activation in Sema 3F KO Mice**. Representative immunohistochemical staining showed increased microglial activation in Sema 3F KO mice, i.e., an increased number of Iba-1 positive cells with enlarged soma and thicker processes (black arrows), compared to their wild type (WT) littermates; *n* = 2.

In agreement with our previous publication, the average staining intensity of Iba1 per unit area in Sema 3F KO mice brain was significantly higher when compared to control mice [17]. In this study, we also noticed morphological changes of microglia in Sema 3F mice compared to their WT control mice, with enlarged soma and thicker processes, characteristic of the phenotypic changes seen in activated microglia (Figure 5).

**Figure 6:**
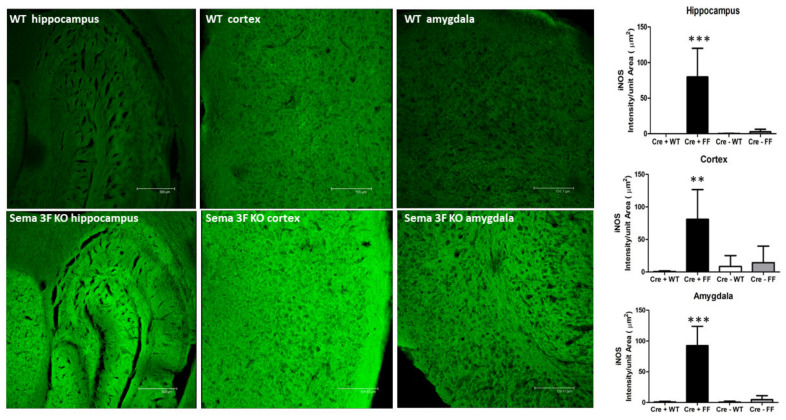
**iNOS Expression in Sema 3F KO Mice**. **Left** panel: iNOS immunofluorescence in hippocampus, cortex, and amygdala of Sema 3F KO mice and their wild type (WT) littermate showed significantly increased iNOS expression in Sema 3F KO tissue. **Right** panel: quantification of iNOS immunofluorescence showed significantly higher fluorescence/unit area in the hippocampus, cortex, and amygdala of Sema 3F KO mice compared to all three control mice; *** *p* ≤ 0.0001; ** *p* ≤ 0.001; *n* = 5.

**Figure 7:**
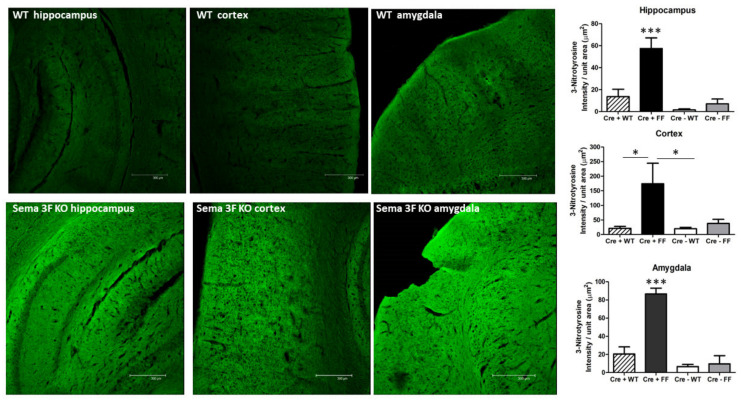
**Induction of 3-Nitrotyrosine in Sema 3F KO Mice**. **Left** panel: 3-Nitrotyrosine (3-NT) immunofluorescence in hippocampus, cortex, and amygdala of Sema3F KO mice and their wild type (WT) littermate showed significantly increased 3-NT expression in Sema 3F KO tissue. **Right** panel: quantification of 3-NT immunofluorescence showed significantly higher fluorescence/unit area in the hippocampus, cortex, and amygdala of Sema 3F KO mice compared to all three control mice; *** *p* ≤ 0.0001; * *p* ≤ 0.01; *n* = 5.

Inflammation and ensuing protein nitrosylation, detected by inducible nitric oxide (iNOS) and 3-nitrotyrosine (3NT) immunoreactivity, significantly increased in Sema 3F KO mice (CRE+FF) compared to control groups (CRE-FF, CRE + WT and CRE-WT), as shown in Figure 6 and Figure 7. A quantitative assessment of immunostaining showed that the average iNOS and 3NT staining intensity per unit area in brain areas of Sema 3F KO mice was significantly higher in the hippocampus (*** *p* ≤ 0.0001; *n* = 5), the cortex (** *p* ≤ 0.001 and * *p* ≤ 0.01 respectively; *n* = 5), and the amygdala (*** *p* ≤ 0.0001; *n* = 5), when compared to the control group. Overall, these results demonstrate that disruption of NRP2-Sema 3F signaling results in widespread cerebral inflammation and oxidative stress in all three brain areas examined in this study.

#### 3.2.3. Immunohistochemical Analysis of CD61 and P-Selectin

**Figure 8:**
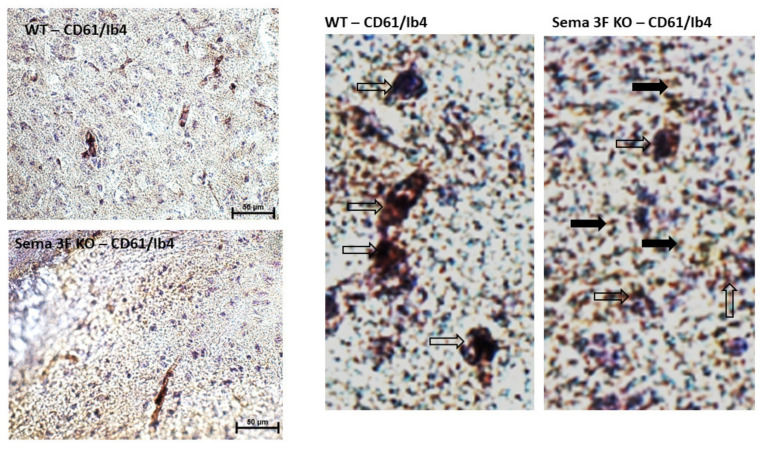
**Localization of Platelets in Sema 3F KO vs. Wild Type Mice**. **Left** panel: Representative immunohistochemical staining of platelet (CD61; brown), vascular (Ib4; purple), with blue hematoxylin counterstain, in brain cortex from Sema 3F KO mice and their littermate wild type (WT) mice. The higher magnification of pictures shown in left panel indicate CD61 immunostaining mostly colocalizing with IB4 in WT mice. In contrast, Sema 3F KO tissue showed CD61 immunoreactivity partly colocalizing with IB4 (clear arrows) and partly out of IB4 (black arrows). The results show a significant increase in platelet infiltration in Sema 3F KO mice compared to more localized vascular platelet staining in the control WT mice.

Given the putative role of vascular inflammation in human-associated autism deficits, we next examine the role of platelet, other endovascular components, and endothelial cell dysfunction. Immunoreactivity of CD61, an integrin β-3 expressed on platelets, significantly increased in the CA1 region of hippocampus of Sema 3F KO mice compared to the control mice, (*** *p*≤ 0.0001; *n* = 3–5). Double staining of CD61 (brown) with the isolectin IB4 (purple), used as a vascular stain, showed a significant increase in platelet infiltration in the cortex of Sema 3F KO mice compared to more localized vascular platelet staining in the control WT mice (Figure 8). Indeed, higher magnification shows that in the WT cortex, CD61 brown staining mainly co-localizes within the purple IB4 staining (clear arrows) while the sema 3F knockout tissue shows CD61-positive cells both within the IB4 staining (clear arrows) and outside it (black arrows).

**Figure 9:**
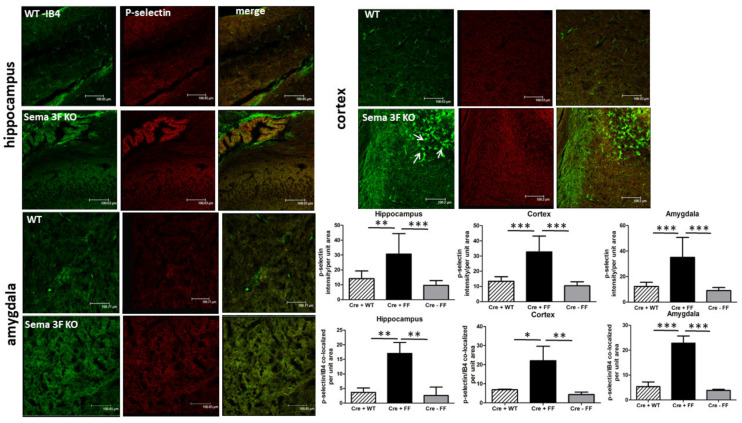
**Co-Localization of Activated Microglia and Platelets in Sema 3F KO Mice**. Representative immunofluorescent staining of p-selectin (red) and isolectin B4 (green) in hippocampus (**upper left**), cortex (**upper right**), and amygdala (**lower left**) tissue from Sema 3F KO mice and their littermate wild type (WT) mice. White arrows show IB4-positive activated cortical microglia. Quantifications of p-selectin (upper part of the lower right panel) showed a significant increase in p-selectin immunoreactivity of Sema 3F KO mice *** *p* ≤ 0.0001; ** *p* ≤ 0.001; *n* = 3–5 and increased p-selectin-IB4 co-localization (bottom part of the lower right panel) in all three brain areas of Sema 3F KO mice compared to the two control mice groups. *** *p* ≤ 0.0001; ** *p* ≤ 0.001; * *p* ≤ 0.01; *n* = 3–5.

Immunofluorescence staining for p-selectin (red), an adhesion molecule expressed at the surface of activated platelets and mediating their adhesion to endothelial cells and neutrophils, showed significantly increased immunoreactivity in the hippocampus *** *p* ≤ 0.0001; ** *p* ≤ 0.001; *n* = 3–5), cortex (*** *p* ≤ 0.0001; *n* = 3–5) and amygdala (*** *p* ≤ 0.0001; *n* = 3–5) of Sema 3F KO mice (CRE+FF) when compared to their control mice (CRE + WT and CRE-FF) (Figure 9). Co-staining with IB4 (green), a lectin vascular stain that has also been shown to mark microglia, shows increased co-localization in all three brain areas (*** *p* ≤ 0.0001; ** *p* ≤ 0.001; * *p* ≤ 0.01; *n* = 3–5). Of note, IB4 staining revealed microglia without radial processes, and was therefore morphologically suggestive of activated microglia in Sema 3F KO t but not in WT cortex (Figure 9). These data strongly suggest increased platelet activation and confirm our observation of increased microglial activation, as shown in Figure 4, in Sema 3F KO mice brains compared to control animals.

#### 3.2.4. Immunofluorescence Analysis of Serotonin Expression

**Figure 10:**
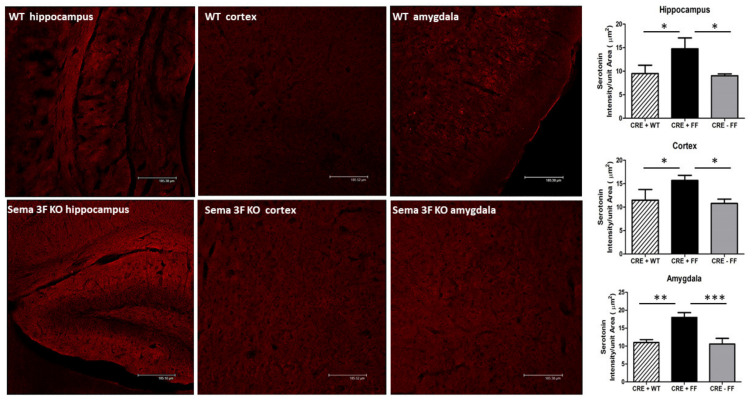
**Elevated Serotonin Expression in Sema 3F KO Mice**. **Left** panel: Serotonin immunofluorescence in hippocampus, cortex, and amygdala of Sema 3F KO mice and their wild type (WT) littermate showed significantly increased serotonin expression in Sema 3F KO tissue. **Right** panel: quantification of serotonin immunofluorescence showed significantly higher fluorescence/unit area in the hippocampus, cortex, and amygdala of Sema 3F KO mice compared to the two control mice. *** *p* ≤ 0.0001; ** *p* ≤ 0.001; * *p* ≤ 0.01; *n* = 3.

Platelets granule release upon activation is a major source of serotonin. Immunostaining of serotonin showed increased and widespread serotonin expression in all brain areas of Sema 3F KO mice compared to a less diffuse and less intense staining in WT animals. A quantitative assessment of serotonin expression in all three brain areas of Sema 3F KO mice (CRE+FF) showed a significant increase when compared to their control groups (CRE + WT and CRE-FF). The average serotonin staining intensity per unit area in the hippocampus, cortex, and amygdala was significantly higher than in the control groups (*** *p* ≤ 0.0001; ** *p* ≤ 0.001; * *p* ≤ 0.01; *n* = 3). (Figure 10). These findings suggest that activated platelets in Sema 3F KO mice brain may release their granule content and contribute to the increase in serotonin expression.

#### 3.2.5. Immunofluorescence Analysis of Fibrinogen and CD31

**Figure 11:**
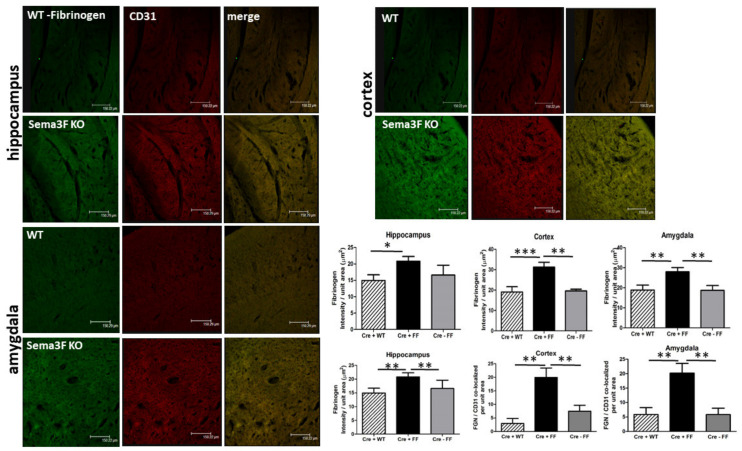
**Expression of Fibrinogen and PECAM-1 in Sema 3F KO Mice**. Immunofluorescent staining of fibrinogen (green) and PECAM 1 (CD31; red) in the hippocampus (**upper left**), cortex (**upper right**) and amygdala (**lower left**) of Sema 3F KO mice and their wild type (WT) littermate showed significantly increased fibrinogen (green) accumulation, co-localizing with CD31 (red) in Sema 3F KO tissue, suggesting vascular inflammation. Quantification of fibrinogen immunofluorescence (upper part of the lower right panel) showed significantly higher fluorescence/unit area in the hippocampus, cortex, and amygdala of Sema 3F KO mice compared to the two control mice. *** *p* ≤ 0.0001; ** *p* ≤ 0.001; * *p* ≤ 0.01; *n* = 3. Quantification of fibrinogen-CD31 co-localization (bottom part of the lower right panel) showed increased co-localization in the hippocampus, cortex, and amygdala of Sema 3F KO mice compared to the two control mice. ** *p* ≤ 0.001; *n* = 3.

Fibrinogen is a glycoprotein that binds platelets αIIbβIII receptor, accumulating during tissue and vascular injury and promoting activated platelet aggregation and clot formation. An immunohistochemical analysis of Fibrinogen and CD31 (Platelet endothelial cell adhesion molecule, PECAM-1) immunoreactivity showed a significant increase of fibrinogen (green) accumulation and infiltration in the brain areas of Sema 3F KO mice (CRE+FF), with clear CD31 (red) co-localization when compared to their control groups (CRE + WT and CRE-FF) (Figure 11). While a trend of increased CD31 was noticeable in all three brain areas, it did not reach significance. Interestingly, CD31 (Figure 11) and P-selectin antibodies (Figure 9) produce little labeling of cerebral blood vessels in WT animals consistent with previous reports in adult mice [39,40]. The average Fibrinogen staining intensity per unit area in the hippocampus, cortex, and amygdala was significantly higher in the Sema 3F KO mice when compared to the control groups, (*** *p* ≤ 0.0001; ** *p* ≤ 0.001; * *p* ≤ 0.01; *n* = 3) (Figure 11). Quantifications of high magnification immunofluorescent merged images shows increased fibrinogen-CD31 co-localization in all three brain areas in Sema 3F KO mice compared to their controls (** *p* ≤ 0.001 CRE +FF vs. CRE + WT and CRE-FF) (Figure 11). These findings strongly suggest that increased platelet endothelial interaction could potentiate vascular inflammation and endothelial injury.

#### 3.2.6. Immunofluorescence Analysis of Albumin Leakage

**Figure 12:**
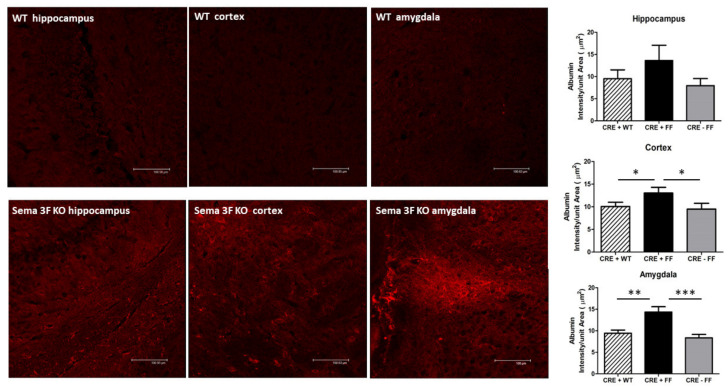
**Vascular Leakage of Albumin in Sema 3F KO Mice**. **Left** panel: Albumin immunofluorescence in hippocampus, cortex, and amygdala of perfused Sema 3F KO mice and their wild type (WT) littermate. While perfusion removed most of the vascular albumin from WT tissue, significant albumin remained in the tissue of Sema 3F KO mice, suggesting vascular leakage. **Right** panel: quantification of albumin immunofluorescence showed significantly higher fluorescence/unit area in the hippocampus, cortex, and amygdala of Sema 3F KO mice compared to the two control mice, which did not reach significance in the hippocampus. *** *p* ≤ 0.0001; ** *p* ≤ 0.001; * *p* ≤ 0.01; *n* = 3.

Inflammation and platelet–endothelial aggregation induce changes in vascular permeability, resulting in trans-vascular albumin leakage. Immunofluorescence analysis of albumin localization showed a significant increase in all three brain areas of Sema 3F KO mice (CRE + FF) when compared to their control groups (CRE + WT and CRE-FF). The staining was present throughout the brain areas and was especially noticeable in the hippocampus, cortex, and amygdala and in the subventricular zone. In some areas, staining appeared as individual cells (neuronal-like morphology), while in others, when cells clumped together, it had a patchy appearance, and in the subventricular zone, it was spread out throughout the ventricular space. The average albumin staining intensity per unit area in the hippocampus showed a clear trend of increased leakage in CRE + FF group but did not reach significance. The average albumin staining intensity per unit area in the cortex and amygdala was significantly higher in the Sema 3F KO mice when compared to the control groups *** *p* ≤ 0.0001; ** *p* ≤ 0.001; * *p* ≤ 0.01 (Figure 12). These results are strongly suggestive of vascular permeability changes that occur with BBB disruption.

## 4. Discussion

Neuroinflammation and microglial activation in the brain is well documented in both postmortem and genomic studies, as well as in animal studies of ASD [2,3,4,13]. Here, we present evidence that parental questionnaires and Vineland Behavioral scales can identify associations with gene variants relevant to RDoC neural circuits. Interestingly, many of these gene variants are classified into vascular signaling systems. These data suggest that human gene variants of neurovascular signaling influence the RDoC neural circuits which are responsible for autism-associated behaviors. In agreement with these findings in humans, we observed ASD-like behavior characteristics as well as morphological changes in iba1- positive microglia, suggestive of microglial activation and neuroinflammation in the brain of Sema 3F KO mice when compared to their control groups [17,41]. Our findings show higher iNOS and 3-NT expression that may result from persistent endothelial and microglial activation. Sema 3F KO brains also displayed clear signs of platelet activation with increased p-selectin, fibrinogen, and serotonin immunoreactivity and endothelial damage with albumin leakage, indicative of BBB disruption. While a few studies have reported platelet involvement associated with ASD, to our knowledge, this study is the first showing signs of platelet activation, endothelial injury, and a breach of the BBB in a murine genetic model with ASD-like behaviors. Given these findings, one of the possible mechanisms involving human neurovascular signaling gene variants may involve BBB integrity and thereby impact the structure/function of RDoC neural circuits.

### 4.1. Development of Blood Brain Barrier and Relationship to Autism Spectrum Disorder

The BBB serves as the interface between the central nervous system and the vascular compartment, maintaining CNS homeostasis [42,43,44,45,46,47,48]. Various pathologies involving neuroinflammation can result in BBB failure [26,44,45,49,50,51,52,53,54]. BBB integrity may also be affected by alterations of gut microbiota and increased gut permeability, as reported in ASD patients [27,55]. This dysfunction allows inflammatory mediators, bacteria, and xenobiotics to pass into the blood stream, contributing to neuroinflammation in ASD patients [27]. Microbiome alterations may also be associated with gastrointestinal disturbances, frequently reported in ASD [28,55]. These variations may be caused by genetic and environmental factors or infections. Microbiota transfer has been shown to decrease BBB permeability [56] and to improve gut–brain axis dysfunction and ASD symptoms [27,55]. BBB dysfunction can also potentially result from genetic abnormalities during development. For instance, alterations in the Sonic Hedgehog (shh), wnt, and beta (β)-catenin signaling pathways during development can result in a dysfunctional BBB and may be associated with ASD pathogenesis [42,48,57,58,59,60,61,62,63]. Indeed, the Panther pathways analysis in this study identified significant shh pathway alterations in ASD subjects (Figure 1B). One of the questions explored in this study was whether developmental neurovascular signaling could regulate blood brain integrity, thereby influencing the formation and output of the RDoC Neural circuits which are responsible for autism-associated behaviors.

### 4.2. Neurogenesis and Vasculogenesis Share Similar Biological Pathways

Developing vascular networks is critical for the migration of GABAergic neurons and their biological function [12,64]. Impaired perivascular GABA release during development is associated with seizures and ASD behavior and affects angiogenesis, neurogenesis, and neuron migration [12,15]. Similarly, the semaphorin-neuropilin system, another neurovascular signaling system, impacts excitatory and GABAergic cell development and vascular development, and its dysfunction results in seizures and autistic behaviors [15,16,42]. Furthermore, in this current study, it was found that both individual SFARI genes (MTOR, BRAF), SFARI listed neurovascular signaling pathways (MAP kinase, B Cell Receptor, and interleukin signaling), Hedgehog signaling, and angiogenesis pathways may influence adaptive, motor, and anxiety behaviors in autism patients (Table 2 and Table 3, Figure 1, Figure 2, Figure 3 and Figure 4). Shh signaling is required for the expression of NRP2-mediated cardiac morphogenesis and induces Sema 3F repulsion of commissural axons during midline crossing of fibers in the spinal cord [65]. Sema 3F is known to regulate glycogen synthase kinase 3 and β-catenin through NRP2 signaling [66,67,68,69]. Shh mediates protective pathways in stroke, regulating microglia/macrophages and inflammatory cytokines, and has been implicated in the BBB response to neuroinflammation [57]. Similar to GABA, neurovascular signaling systems such as the sema 3F-NRP2 system are highly expressed both in neurons and endothelial cells [16,70]. Their interactions and the biophysical and behavior properties of those interactions have not been investigated in the context of ASD. This pilot study explores the interplays and regulatory mechanisms which are potentially involved.

### 4.3. Semaphorin 3F Biology and the Blood Brain Barrier

Class III Semaphorins have been the subject of a fair number of studies, but none has extensively studied Sema3 F-NRP2 signaling and its role in the BBB integrity. BBB dysfunction has been reported with several neurological disorders [26,49,50,51,53,71]. In a postmortem study of ASD patients, the functional integrity of BBB proteins was altered, suggesting a dysfunctional BBB [26]. BBB breach was also demonstrated in a rat prenatal valproic acid model of ASD [72,73]. Interestingly, our data showed albumin leakage and accumulation within neurons throughout the brain tissue, including significant increases in cortex, hippocampus, and amygdala in Sema 3F KO mice (Figure 12). Therefore, the Sema 3F KO model of autism and epilepsy is the first documented ASD genetic model to show strong and compelling evidence of BBB vascular breach. While there are reports on gut–brain barrier dysfunction in ASD [74,75], to the best of our knowledge, no previous study has documented or investigated the role of BBB disruption and cellular mechanisms in a mouse model of neurovascular signaling associated with autism-like behaviors.

### 4.4. Neuroinflammation in Autism Spectrum Disorder

Studies using human brain samples suggest that children with ASD show evidence of microglial activation and ongoing neuroinflammatory processes in different brain regions [2,4,76]. However, normative data from large numbers of neurotypical children are not available to further delineate the line between adaptive and maladaptive processes. Microglia play an important role in neurogenesis, postnatal apoptosis, synaptic connections, etc., [77,78,79,80]. Microglial activation, shown here by enlarged soma and thicker processes (Figure 5), can also generate inflammatory mediators, reactive oxygen species, and reactive nitrogen species, as evidenced in the current study (Figure 6 and Figure 7). The growth of developing brain may be altered by the presence of oxidative stress and ongoing inflammation in autistic children [73,81,82,83]. Indeed, we found lower brain weights in our Sema 3F KO ASD mouse model, DLX5/6^Cre^ KO mice (Table 4). In a study comparing mouse lines selectively bred for smaller brain to mice bred for larger brain, the mice with a larger brain displayed more successful learning, while the smaller brain mice displayed more fear and anxiety, higher excitability and were more active (locomotion and exploration), more sensitive to stress and to pain [84,85]. Another study reports different brain weights in Balb/c mice raised in a similar environment for 5 months, that the authors attributed to social factors. However, similarly to Markina et al., they found that smaller brain weight was associated with increased activity and exploration [86].

Microglial cells are deeply reactive to their environment, altering their morphology to adapt to their microenvironment, neuronal activity, and synaptic signaling changes [87,88,89,90,91]. Several studies have attempted to link neuroinflammation and microglial changes with pathophysiological characteristics of mouse models of brain disease [89,92]. BTBR mice, an inbred mouse model of ASD, exhibit a neuroinflammatory profile that correlate with some of their repetitive behavior, characteristic of ASD [93,94]. Impaired neuronal function as a result of microglial activation and inflammatory processes could lead to the EEG changes we identified in DLX5/6^Cre^ CRE+FF mice (Table 5). Although EEG is widely used to detect epileptic activity, increased cortical excitability may contribute to EEG abnormalities in ASD patients, even without the occurrence of seizures [95,96]. EEG diagnostic values for ASD, along with various analysis methods, have recently been considered (reviewed in [97]). In particular, lower alpha frequency spectrograms have been reported in ASD patients [98]. A recent study reported that EEG measurements can identify ASD and predict its severity in children as young as 3 months old [99]. In light of these reports, the EEG changes and lower brain weight detected in our current study in DLX5/6^Cre^ Sema 3F KO mice agree with our previous report of increased epileptogenesis and autistic behavior in these mice, while EMX1^Cre^ Sema 3F KO mice exhibited no differences in brain weight and had no evidence of epileptogenesis [17].

### 4.5. Interactions between Semaphorin and Immune Signaling

The activation of inflammatory factors released by microglia may exacerbate continued pathological processes [100,101]. Increased iNOS and 3NT expression in ASD-relevant brain areas of Sema 3F KO, such as cortex, hippocampus, and amygdala, are characteristic of microglial activation [100,101] and strongly suggest oxidative stress and inflammatory response in key neural circuits relevant to ASD. These processes could result either from the lack of Sema 3F action on interneurons/pyramidal cells or from the lack of Sema 3F regulation of related microglial activity. Previous studies also presented an autistic behavioral phenotype consistent with ASD in both the Sema 3F and the NRP 2 KO mice [16,17,22]. Both secreted and membrane bound semaphorins can contribute to the polarization and inflammatory state of macrophages and presumably microglia, which expressed NRP2 [102,103,104]. It is intriguing to think that a lack of proper Sema 3F signaling may contribute to microglial dysfunction and neuroinflammation, thereby affecting neural development in autism [2,3,4]. The association of gene variants involved in inflammation and human autism behaviors (Table 2 and Table 3, Figure 1, Figure 2, Figure 3 and Figure 4) further reinforces this intriguing possibility.

The endothelium plays an important role in the inflammatory response as a key regulator of vascular permeability during inflammation [42,105,106,107,108,109,110,111]. Mucka et al. reported that NRP2 deficiency enhanced blood vessel permeability during inflammation [111], in agreement with our data in the Sema 3F KO. iNOS, which was originally identified in platelets and inflammatory cells, is widely distributed in the endothelium and vascular smooth muscle. Upon inflammatory conditions, fibrinogen is upregulated, recruiting inflammatory cells and platelets, activating endothelial cells, and contributing to persistent vascular inflammation causing platelet aggregation and vascular leakage [106,109,112,113]. Fibrinogen, a main component of blood clots and essential to vascular homeostasis, is normally excluded from the brain parenchyma by the blood brain barrier. However, neurovascular damage can allow fibrinogen access to the central nervous system and further worsening the pathological conditions. It was earlier reported that fibrinogen is present in the brains of neurodegenerative patients accumulating in neurovasculature and attracting more platelets and microglia [114,115,116]. Although CD31 increase did not reach significance, its accumulation in proximity of fibrinogen strongly suggests endothelial inflammation with increased platelet–endothelial adhesion in the cortex, amygdala, and hippocampus of the Semaphorin 3F knockout mice (Figure 11). In support of this hypothesis, Class III semaphorins such as Sema 3F are known to affect endothelial cell migration, angiogenesis, and monocyte/neutrophil recruitment to sites of inflammation [70,117]. Importantly, both physiologic factors and inflammatory mediators (TNFα, IL-1β) regulate the expression of sema 3F in endothelial cells and sema 3F itself regulates both the barrier function and adherens junctions in endothelial cells [70]. These data suggest that neuronal derived semaphorin could affect the function of blood brain barrier (BBB) and allow plasma proteins to leak into the brain parenchyma.

### 4.6. Semaphorin and Serotonin Signaling

Vascular inflammation and oxidative stress alter BBB integrity. Exposed endothelial cells can anchor platelets to the subendothelium and activate them to release inflammatory granule content and serotonin, escalating adhesion and the inflammatory process [43,118,119]. Platelet–endothelial activation has been reported in neurological diseases [120,121] and in ASD patients [122,123], but little is known about the details of such activation. Furthermore, Sema 3F is known to inhibit adhesion and cell motility in endothelial cells [124]. Thus, Sema 3F deletion in our mouse model could underlie increased platelet–endothelial association (Figure 8). Semaphorin signaling in platelets may regulate biological processes including thrombus formation in a contact dependent manner [125]. Strikingly enough we have uncovered a significant enhancement of activated platelets in the brains of ASD mice when compared to the wild type mice (Figure 8 and Figure 9) with concomitant high level of serotonin and fibrinogen in hippocampus, cortex, and amygdala in the ASD mice (Figure 10 and Figure 11). The serotonergic system has been implicated in ASD etiology [126]. Elevated blood serotonin, one of the first biomarker identified in autism spectrum disorder, occurs in excess of 25% of affected children, and the percentage release of serotonin itself can be used as a marker to assess the level of platelet activation [127,128,129]. All of these findings are likely to emanate mostly from activated platelets within the brain tissue itself. Indeed, while both CD31 and p-selectin are expressed in endothelial cells and platelets, both are critical to platelet–endothelial interactions and the increased p-selectin expression which occurs at the time of platelet activation, recruiting flowing leukocytes and contributing to inflammation [112]. P-selectin and CD31/PECAM-1 labeling was mainly observed in cerebral tissue rather than in blood vessels (Figure 9 and Figure 11) [39,40]. Similarly, soluble P-selectin and CD31/PECAM-1 are decreased in the plasma of children with ASD compared to age/sex matched neurotypical controls [130].

Serotonin has been linked to developmental processes due to its various roles across multiple brain systems, both dynamically and across development. The serotonin system is a logical candidate for involvement in ASD, but its contribution to ASD pathophysiology remains incompletely understood. Serotonin is actively taken up and most of the peripheral serotonin is stored by platelets to be released with inflammatory granule content upon activation, thereby further increasing platelet activation and serotonin release in the brain [131]. A recent study modulating serotonin levels by dietary restriction of its precursor tryptophan or by using serotonin transporter knockout mice showed that lowering serotonin levels normalizes behavioral deficits relevant to ASD [132]. Markers of platelet activation, elevated platelets, and serotonin levels have also been reported in ASD patients. In fact, gender specific effects of serotonin signaling may impact threshold levels of ASD phenotypes [133]. Several reports suggest that serotonin, acting thru 5-HT2B receptor, likely controls the M1 (inflammatory) vs. M2 (anti-inflammatory) status of microglia, thereby impacting synaptic development/numbers, and altering synaptic plasticity/excitatory neurotransmission [128,134,135,136]. Such alterations may promote autism-like behaviors from diverse ASD-relevant areas including somatosensory cortex and cortico-striatal circuits [137,138]. Given the interactions between serotonin, inflammation, microglia, and gender during various developmental brain stages, platelet activation and serotonin release in distinct brain epochs may play a key role in the pathophysiology of neuroinflammation in ASD [122,123,128,134].

### 4.7. Impact of Impaired Developmental Neurovascular Signaling on CNS Development and Neuroinflammation

BBB development during embryogenesis is a gradual process, beginning with vascularization of the brain from the perineural vascular plexus at approximately E9 and initiated by the release of growth factors from the neural tube. CNS blood vessels come in contact with astrocytes and pericytes and acquire functional tight junction (TJ) proteins, forming a network, and starting to express various transporters to form the BBB [139,140]. The Wnt/β-catenin signaling pathway is critical during angiogenesis and barriergenesis [42]. Shh, released by astrocytes, signals to endothelial cells to induce Wnt/β-catenin signaling, forming TJ proteins and junctional adhesion molecules. The Shh signaling pathway also suppresses inflammatory mediators and inhibits infiltration of immune cells into the CNS [47,57,141]. Shh/Wnt pathway and vascular systems crosstalk control neurovascular unit development and regulate neural progenitor cells differentiation, neuronal migration, axon guidance, dendritogenesis, synaptogenesis, and BBB formation/function. Many of genes identified in our ASD human data and Sema 3F-NRP2 signaling are downstream of these important BBB developmental and CNS maintenance mechanisms. These pathways also play a role in immune modulation and in the resolution of inflammation [142]. The Neuropilin/class III semaphorins system has been shown to be critical to embryonic angiogenesis [143,144]. In a model of acute inflammation, NRP-2 deficiency increased vascular permeability and decreased lymphatic capillaries, resulting in severe swelling and lymphedema, and addition of Sema 3F prevented increased vascular permeability and loss of lymphatic drainage, critical to limiting inflammation [107,111]. Furthermore, our studies, using the Sema 3F KO mouse model, uncover vascular injury, with increased p-selectin, fibrinogen, serotonin, and albumin leakage, suggestive of platelet activation and disrupted BBB (Figure 9, Figure 10, Figure 11 and Figure 12). Sema 3F has also been shown to prolong platelets lifespan, and increased platelet counts have been reported in ASD that may enhance platelet aggregation and endothelial injury, compromising BBB integrity [123,145]. During activation, platelets alpha granules release several important proteins such as fibrinogen, P-selectin, von Willebrand factor, growth factors, while dense granules release glutamate and interestingly serotonin, one of the first ASD biomarkers identified [146,147,148]. Overall, neurovascular signaling may not only affect CNS development, neurite growth, and axonal guidance, but may also dysregulate immune and inflammatory responses and vascular development, all potentially contributing to BBB dysfunction, thereby impacting neural circuits and behavioral outputs.

### 4.8. Limitations

**Figure 13:**
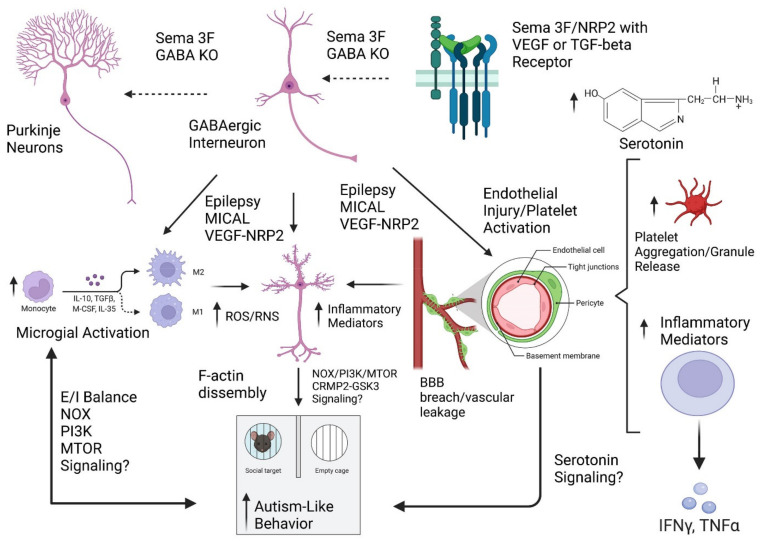
**Schematic Representation of Experimental Hypothesis**. Neurovascular molecules involved in neurodevelopment such as Sema 3F-NRP2 signaling are expressed in microglia, endothelial cells, as well as GABAergic neurons such as cortical interneurons or Purkinje cells. Altered signaling (dashed lines) of neuronal derived target vascular molecules may influence the BBB integrity or function, such that compromised barriers can lead to an influx and generation of inflammatory mediators, ROS/RNS, microglial activation, and platelet dysfunction. Thus, the altered impact of either PI3K-MTOR, altered E/I balance, cytoskeletal, or serotonin pathways can lead to abnormal formation and function of ASD relevant neural circuits, promoting autism-like behaviors.

Our initial pilot study is one of the first to examine the relationship between the developmental expression of gene variants, RDoC neural circuits, neurovascular inflammation, and autism-like behaviors. Although each of these neural circuits is dysfunctional in autism, the dysfunction of these neuroanatomical pathways is not specific to autism. The overall structural mapping of cognition and behaviors to distinct neuroanatomical and functional linked neural circuits is likely to server not only in diagnoses but also in the mapping of a cluster of ASD individuals whose behaviors and characteristics are more similar than different. However, our understanding of gene expression in normal human brain development, its change with neurodevelopmental trajectories such as autism, and how that impacts neural circuit structure and function are poorly understood. Further, genetic and anatomical variation can only suggest hypotheses in the face of (1) limited clinical phenotyping and (2) a limited number of humans with defined genetic variants. Furthermore, we did not investigate cell types and mechanisms in humans, but rather, in a more tractable animal model. A limitation of our conclusions from animal data is that it is unclear which cell type is regulated by Sema 3F other than excitatory pyramidal cells [25,149], and NRP2 receptors are expressed on a variety of cells [103,104]. Furthermore, the localization of downstream signaling systems including PI3K-Akt-mTOR/Nox [124], as well as the possibility that some of this pathophysiology emanates from the aberrant regulation of the MICAL pathway downstream of the NRP2 receptor, should be considered [150,151,152]. Therefore, future studies will identify the cell types sensitive to and processes regulated by Sema 3F-NRP2 in our animal models, that could regulate neural circuits responsible for autistic behavior, and whether other neurovascular molecules mutations potentiate these effects in mice and predispose to ASD in humans. Furthermore, the impact of Sema 3F-NRP2 signaling on the VEGF-ERK system during brain development remains to be explored [153]. ERK knockouts, human CNV deletion syndromes (22q11.2 and 16p11.2) and pharmacological blockage of ERK signaling during a critical period, results in neuronal/glial apoptosis and autistic behaviors [154]. Analogies with work in humans are hard at this point, due to the scarcity of literature and to the small and inhomogeneous samples inherent to available studies. Nevertheless, our pilot study establishes the need to investigate neuroinflammatory and vascular processes in the context of ASD/developmental disorders in humans and provides a novel murine genetic model suitable for these studies. The signaling pathways that could potentially be implicated in the pathological processes described in this study that will be explored in the future are indicated in Figure 13.

## 5. Conclusions

Shh/Wnt signaling and other developmental autism loci may regulate the expression of neurovascular signaling, such as the Sema 3F-NRP2 system during brain development, and thus, may represent a set of novel downstream therapeutic targets. As Shh/Wnt signaling is required for angiogenesis and BBB formation, there is a high likelihood of crosstalk between such autism developmental loci and neurovascular signaling (like Sema-NRP pathways). These are novel examples of potential interactions between neuronal-based and endothelial-based cell signaling which affects brain development (Figure 13). In this regard, one can see parallels between inflammatory processes in autism and another genetically related developmental disorder, schizophrenia (SCZ) [155,156] In SCZ, inflammatory markers are associated with cortical expansion and impaired blood brain barrier function which correlates with symptom severity [157,158]. Using human induced pluripotent stem cells, SCZ-derived brain endothelial cells have decreased vasculogenesis and permeability, and decreased ZO-1 expression (tight junction protein) with altered cellular localization in the cytoplasm [159]. These data suggest that both the expression of gene variants of signaling pathways (protein kinases C, G, and A, RhoA, PI3/Akt, and Wnt) which control BBB function in patients and investigation of the disrupted pathways in autism may yield druggable targets [160]. Interventions targeting specific regulators of these pathways rather than the whole pathway, either during development or at later stages, will also allow more selective outcomes, avoiding disruption of the normal development and aiming at ASD treatment and maintenance of the CNS. These and other novel biologicals targeting the regulation of neurovascular pathways or downstream targets could alleviate some ASD symptoms.

## Figures and Tables

**Table 1:** Number of Autism Subjects with Questionnaires per Experimental and Control Groups.

Number	PCQ (N)	Vineland (N)
Group 1 (Normal CMA)	47	30
Group 2 (CNVs)	90	87
total	137	129

**Table 2:** Associations Between Gene Variants, Neural Circuits, and Behaviors.

Behavior	Circuit	Group	Number	Mean	Std. Error	*p*	Brain Region	Related Genes
PCQ12- Self-stimulatory behavior	Inter-group	1	47	2.79	0.158	0.037		
		2	90	2.39	0.107			
PCQ3-Anxiety	Positive Valence system	1	129	2.57	0.090	0.006	MPFC, ACC, Amy, STR	COG5, AKT1, PDHA1, CHEK2 *, RARS *; PIAS1 *; CTDP1
		2	13	2.00	0.160			
	Cognition system	1	121	2.59	0.091	0.029	ACC, Ocx, DLPFC, MD, A1C, IPC, STR, V1C,	COG5, AKT1, PDHA1, CHEK2 *, RARS *; BRAF *, FANCI *, COG2 *; OLD *; FAN, GRIN2A; CREBBP, ORC4, IKBKG; CTDP1; FANCL;
		2	21	2.10	0.194			
PCQ3-Anxiety	Social communication system	1	124	2.58	0.090	0.042	IPC, ACC, Ocx, ITC, V1C, A1C, STR	COG5, AKT1, PDHA1, CHEK2 *, RARS *; CREBBP, ORC4, IKBKG, IRS1; BRAF *; EIF2B3; FANCL; GRIN2A; CTDP1;
		2	22	2.14	0.190			
Vineland ADL(Daily Living Skill)	Positive Valence system	1	111	68.26	1.110	0.028	STR, ACC,	PEX14, CTDP1, RARS *; RARS *, COG5, CHEK2 *, RNASEH2C, PDHA1;
		2	6	76.00	2.582			
Vineland Motor	Positive Valence system	1	111	74.71	1.183	0.030	STR, ACC,	PEX14, CTDP1, RARS *; RARS *, COG5, CHEK2 *, RNASEH2C, PDHA1;
		2	6	86.00	3.044			
Vineland Motor	Cognition system	1	104	74.53	1.244	0.025	Ocx, VLPFC, STR, DLPFC, A1C, ACC, IPC	MTOR *, COG2 *, RNASEH2A *; PEX10, MAP2K2; FEX14, CTDP1; EIF2B4, NDUFV2 *; PTPN11; CHEK2 *, PDHA1, RARS *, COG5, RNASEH2C; ORC4, CREBBP, IKBKG;
		2	13	81.38	2.521			

ACC Anterior Cingulate Cortex, Amy Amygdala, STR striatum, OCx occipital cortex, DLPFC dorsolateral prefrontal cortex, MD mediodorsal nucleus of thalamus, A1C primary auditory cortex, IPC inferior parietal cortex, STR striatum, V1C primary visual cortex (area V1/17), ITC inferolateral temporal cortex (area 20), VLPFC ventrolateral prefrontal cortex * PCW between 8–9 weeks.

**Table 3:** Panther Pathways Analysis of Gene Variants expressed in RDoC Neural Circuits.

Behavior	Circuit	Brain Region	Related Genes	Panther Pathways Analyses
PCQ3-Anxiety	Positive Valence system	MPFC, ACC, Amy, STR	COG5, AKT1, PDHA1, CHEK2 *, RARS *; PIAS1 *; CTDP1	PI3K/Akt/mTOR-Vit D3 signaling (*p* = 0.00009)
				Lipid metabolism pathway (*p* = 0.0001)
PCQ3-Anxiety	Cognition system	ACC, Ocx, DLPFC, MD, A1C, IPC, STR, V1C	COG5, AKT1, PDHA1, CHEK2 *, RARS * BRAF *, FANCI *, COG2 *; OLD *; FAN, GRIN2A; CREBBP, ORC4, IKBKG; CTDP1; FANCL;	Estrogen signaling (*p* = 0.000003)
				DNA IR-damage and cellular response via ATR (*p* = 0.0001)
	Social communication system	IPC, ACC, Ocx, ITC, V1C, A1C, STR	COG5, AKT1, PDHA1, CHEK2 *, RARS *; CREBBP, ORC4, IKBKG, IRS1; BRAF *; EIF2B3; FANCL; GRIN2A; CTDP1;	Estrogen signaling (*p* = 0.000005)
				Leptin signaling (*p* = 0.0002)
Vineland ADL(Daily Living Skill)	Positive Valence system	STR, ACC	PEX14, CTDP1, RARS *; RARS *, COG5, CHEK2 *, RNASEH2C, PDHA1;	amino acid metabolism (*p* = 0.001)
Vineland Motor	Positive Valence system	STR, ACC	PEX14, CTDP1, RARS *; RARS *, COG5, CHEK2 *, RNASEH2C, PDHA1	amino acid metabolism (*p* = 0.001)
Vineland Motor	Cognition system	Ocx, VLPFC, STR, DLPFC, A1C, ACC, IPC	MTOR *, COG2 *, RNASEH2A *; PEX10, MAP2K2; FEX14, CTDP1; EIF2B4, NDUFV2 *; PTPN11; CHEK2 *, PDHA1, RARS *, COG5, RNASEH2C; ORC4, CREBBP, IKBKG	Leptin signaling (*p* = 0.000004)
				IL-6 signaling (*p* = 0.00007)

DLPFC dorsolateral prefrontal cortex, MD mediodorsal nucleus of thalamus, A1C primary auditory cortex, IPC inferior parietal cortex, STR striatum, V1C primary visual cortex (area V1/17), ITC inferolateral temporal cortex (area 20), VLPFC ventrolateral prefrontal cortex * PCW between 8–9 weeks.

**Table 4:** Brain and Body Weights of Cell Specific Semaphorin 3F Deletion Mice.

Strain at P21	Body (gms)	Brain (gms)	Brain/Body Ratio
DLX5/6^Cre^			
CRE-FF	10+/−2.0	0.41+/−0.03	0.042+/−0.007
CRE+FF	8.3+/−0.4 0.37+/−0.02		0.045+/−0.004
*p* Value	*p* = 0.14 *p* = 0.05		*p* = 0.39
EMX1^Cre^			
CRE-FF	8.94+/−2.0	0.39+/−0.03	0.046+/−0.009
CRE+FF	8.31+/−1.0	0.39+/−0.01	0.047+/−0.006
*p* Value	*p* = 0.48 *p* = 0.53		*p* = 0.70

**Table 5:** EEG Power of Interneuron Cell Specific Semaphorin 3F Deletion Mice.

Strain at P60-P100	CRE+FF (µV^2^)	CRE-FF (µV^2^)	*p* Value
DLX5/6^Cre^			
Alpha	73+/−9	112+/−16	0.060
Beta	46+/−6	108+/−12	0.009
Delta	203+/−27	221+/−32	0.460
Theta	194+/−23	209+/−41	0.680

## Data Availability

All data supporting the reported results are published within this manuscript.

## Supplementary Materials

The following supporting information can be downloaded at: https://www.mdpi.com/article/10.3390/cells11142211/s1, File: Supplemental Methods; Table S1: List of patients CNVs.
