# Supplementary material for: Potential Cross Talk between Autism Risk Genes and Neurovascular Molecules: A Pilot Study on Impact of Blood Brain Barrier Integrity
Source: Cells. 2022 Jul 15;11(14):2211. doi: 10.3390/cells11142211 (PMC9315816; doi:10.3390/cells11142211)
Supplement: Supplementary file 1 [file cells-11-02211-s001.zip › cells-1748138-supplementary.pdf]

Methods:

IRB Approvals

All studies and access to databases were reviewed and approved by the NIMH and human subject studies institutional review boards of the University of Louisville and Vanderbilt University Medical Center.

Protein-Protein Interactions

The genes derived from the enrichment analysis using WebGestalt were further evaluated for their protein-protein interactions using STRING (Szklarczyk et al., 2015). A confidence level of 0.90 was used to formulate the interactions. STRING combines the probability of associations between proteins, both direct and indirect, from evidence collected across various arenas of databases. The association is calculated based on evidence from experimental data, mining of literature and databases, and genome context analysis (Szklarczyk et al., 2015). These associations are then corrected for the probability of a random observation of an interaction.

Mapping Brain Gene Expression to RDoC Neural Circuits

By integrating the data of these genetic data, the maximum mRNA expression of the gene during pregnancy and post-pregnancy and the brain regions during that period was identified. After obtaining the maximum mRNA expression of the gene (time, brain region), the brain regions associated with each neural circuit were mapped through NIMH's RDoC-matrix (https://www.nimh.nih.gov/research/research-funded-by-nimh/rdoc/constructs/rdoc-matrix.shtml). Through the database, the corresponding circuits were matched to the brain regions with the maximum mRNA gene expression.

The RDoC neural circuits was divided into 1) Negative Valence Systems (including fear, anxiety, sustained threat, loss, frustrative non-reward), 2) Positive Valence Systems (Reward responsiveness, reward learning, reward valuation), 3) Cognitive Systems (Attention, perception, Declarative memory, Language, Cognitive Control, working memory), 4) Social Processes (Social communication, perception and understanding of self, in the loop of RDoC. Perception and understanding of others), Arousal and regulatory systems (arousal, circadian rhythms, sleep-wakefulness), and 5) Sensorimotor systems (Motor actions, agency and ownership, Habit-sensorimotor, Innate Motor patterns). According to these circuits and gene variant as the guide, we map the neural circuits to each subject with a given gene variant in the associated brain regions.

For a given question answered by parents, the scores of those ASD subjects with specific genetic variation in a given neural circuit was compared with ASD subjects with no genetic variation (i.e.- those ASD subjects with normal microarray: average age 11.2 years) in different behavior scales. ASD subjects with specific genetic variation were grouped together within the genetic variation group (Group 1) by 1) specific same gene variation and 2) maximal mRNA expression in a given brain region contained in the RDoC neural circuits. For each PCQ question or Vineland Scales subscore, all RDoC neural circuits in those with genetic variants were compared to scores of those ASD subjects with normal microarrays (Group 2).

Statistical analysis for Neural Circuit Comparisons:

All data were performed using SPSS software11 (IBM SPSS Statistics for Windows, Version 24.0. Armonk, NY: IBM Corp) and plots were generated using GraphPad Prism version 8 (GraphPad software, San Diego, CA, USA). Equality of variances was tested by Levene's Test. Within group comparison was performed for behavioral scores by t-test for equality of variances. In case of inequality of variances, other data were analyzed using two-way ANOVA followed by Tukey post-hoc test. Two tailed Pearson analysis was used to find the correlation between variables. Data were presented as mean ± SEM. The null hypothesis was rejected with $p < 0.05$ as the level of significance.

Mouse Experiments

The animal experiments were performed under protocols approved by the Vanderbilt University and the University of Louisville Institutional Animal Care and Use Committee.

Mouse lines: Semaphorin 3F knockout mice (CRE+FF) [DLX5/6^{Cre}, deletion in GABAergic Neurons] and control mice (Cre−FF, Cre+WT, Cre−WT) lacking either Cre expression or Flox sites flanking the Sema 3F gene, or both, therefore expressing normal levels of Semaphorin 3F, were described in our previous publication (Li et al., 2019), and were used in the current study. 3-5 animals (3 to 6 months old) per group were used for immunofluorescence and immunohistochemical analysis. Animals were anesthetized and perfused transcardially with 4% paraformaldehyde, embedded and

cut into 30-μm thick sections, then mounted onto charged slides. Brain of DLX5/6^{Cre} knockouts and of EMX 1^{Cre} heterozygotes [deletion in excitatory neurons] sacrificed at weaning, were weighed and compared to their respective controls.

Immunofluorescence: Sections were blocked with normal donkey or goat serum for 1 hour and incubated overnight at 4°C with primary antibodies such as iNOS (Abcam : Rabbit ,1:50), 3-NT (Millipore: Rabbit, 1:500), Serotonin (Abcam: Goat, 1:500), Albumin (Bethyl: Goat Polyclonal, 1:500), Fibrinogen (Agilent: Polyclonal Rabbit, 1:200), CD31 (Abcam: Rat, 1:100), p-selectin (Santa Cruz:Mouse 1:50), or with Biotin-conjugate isolectin B4 (IB4 Sigma: 1:200). The sections were incubated with appropriate FITC/TRITC-tagged anti-rabbit/ anti-mouse/ anti-rat or anti- goat secondary antibodies (Jackson ImmunoResearch, West Grove, PA) or with extravidin-FITC (Sigma) for IB4 staining, for 1 hour at room temperature in the dark. The stained sections were then mounted, and the images were captured using a laser scanning confocal microscope (Leica-TCS SL, Wetzlar, Germany) and staining intensity per tissue unit area (μm^2) as well as co-localization in high magnification images, were quantified and averaged in 3 ROIs per slide in 3 slices per animal, using image analysis software (Image-Pro Plus). Data were expressed as intensity per unit area ± SD.

Immunohistochemistry: 30-μm thick sections obtained from cryostat sectioning were blocked with normal donkey serum for 1 hour. Primary antibodies were applied overnight at 4°C Iba1 (Wako Pure Chemical Industries: Rabbit, 1:450), CD61 (Invitrogen: Hamster Anti Mouse,1:200), IB4 (Sigma: Lectin,1:200). The sections were then incubated with appropriate biotinylated secondary antibodies (1:200, Vector Laboratories), followed by avidin/biotinylated enzyme complex treatment (Vectastain Elite ABC Kit, Vector Laboratories), and counterstained with hematoxylin (Vector Laboratories). Proteins were detected on color development using 3,3′-diaminobenzidine as substrate or VIP (Vector Laboratories). The images were captured using a Nikon microscope.

Statistical analysis: GraphPad Prism 6 software (GraphPad Software, San Diego, CA, USA) was used for the statistical analyses. Repeated measures two-way ANOVAs, followed by post-hoc Tukey’s Multiple Comparison Test, were used to compare CRE-FF Sema 3F-KO mice with Cre−FF, Cre+WT, and Cre−WT mice, expressing normal levels of Semaphorin 3F. Data are presented as mean ± SD. Differences between means were considered as significant at $p \leq 0.05$.

Table S1. List of patients CNVs.

Patient ID	Chromosomal location	Genes	Type of mutation	Autism/ Epilepsy	Chromo. Physical Location
V-4009	1p33		copy loss	both	49993471-50241585
V-5004	1p31.1		copy loss	both	83134636-83204648
V-7088	1q42.3		copy gain	autism	235242449-235566242
L-AA-06	1q44		copy gain	autism	248752350-248788599
L-AE-06	1q44		copy loss	both	246818971-246855500
V-4005	2p23.3		copy loss	both	26310934-26320554
V-6002	2p16.1		copy loss	both	57972987-58321855
L-AE-02	2p15		copy gain	both	61339194-61837347
V-7001	2q23.3		copy loss	autism	152518150-152621528
V-4003	2q34		copy loss	both	45468474-51263952
V-4007	2q35		copy loss	both	21559996-21569576
L-AE-15	3p14.1		duplication	both	65925174-66369539
V-7079	3p14.1		copy loss	autism	67662230-69109358
L-AA-06	3q26.1		copy gain	autism	162545622-162619142
V-085	3q29		copy loss	both	197118426-198878855
V-7073	4p16.3-16.2		copy gain	autism	2879238-3143876
V-035	4p14		copy loss	both	40293500-40350912
V-7080	4p12		copy loss	autism	52912914-52949184
V-057	4q22.3		copy gain	both	95971459-96192102
V-7028	4q31.23		copy loss	autism	149335718-149513891
V-7027	5q35.1		copy loss	autism	169270815-169793639
L-AE-02	6p25.3		copy gain	both	211046-321106
L-AA-06	6p25.3		copy gain	autism	303239-375950
V-6001	6p12.3		copy gain	both	RP113427 to RP1166H4
V-7036	6q15		copy loss	autism	88843447-88942270
V-7071	6q23.1-23.2		copy loss	autism	131232705-131361909
V-7038	7p22.2		copy loss	autism	46757237-51207712
V-7052	7q21.3		copy loss	autism	93449581-94035079
L-AE-02	7q22.2		copy loss	both	105690859-105845281
L-AE-02	7q31.33		copy loss	both	125406941-125530771
V-7034	7q31.33		copy loss	autism	126232239-126329405
L-AE-06	8p23.3-23.2		duplication	both	1703070-2285210
L-AA-03	8p23.1		duplication	autism	10105550-10586767
L-AE-33	8p22		copy gain	both	16965962-17073395
V-7081	8p12-11.1		copy gain	autism	32406314-46938968
L-AA-06	8p11.22		copy loss	autism	36372150-36435256
L-AE-33	8p11.21		copy gain	both	39395980-39490567
L-AA-06	8p11.21		copy gain	autism	39246720-39371396
L-AE-02	8p11.21		copy loss	both	39365946-39490567
L-AE-06	8p11.21		copy loss	both	39365946-39490567
V-006	9p21.2-21.3		copy gain	both	25108496-25703810
V-5002	9q21.13-21.32		copy loss	both	67915482-77932017
V-086	10p14		copy gain	both	10775715-11877958
V-7049	10p13		copy loss	autism	15018761-15125968
V-4002	10p11.23		copy gain	both	29526175-29874022
L-AE-33	10q11.1		copy loss	both	46400346-46568287
V-5006	10q11.22-11.23		copy loss	both	45468474-51263952
V-6003	10q11.22-11.23		copy gain	both	45468474-51671987
V-7053	10q11.23		copy loss	autism	52939210-53085688

V-064	10q26.3	copy gain	both	133200689-133769778
V-7050	13q31.3	copy loss	autism	93249840-93301534
V-7087	14q32.13	copy gain	autism	94496673-94836029
V-087	15q11.1-13.1	copy gain	both	20191652-28525505
V-073	15q11.2	copy loss	both	25068625-25091478
V-7068	15q11.2	copy loss	autism	20014155-20851577
V-5005	15q11.2-13.1	copy gain	both	20642827-27938509
V-7074	15q13.3	copy loss	autism	28456615-30247879
V-7081	15q13.3	copy loss	autism	31730445-32438736
L-AE-14	15q13.3-14	copy loss	both	31291942-39213754
V-7038	15q21.1-21.2	copy loss	autism	46757237-51207712
V-6005	16p24.3	copy gain	both	89967950-90085882
V-4006	16p13.11	copy loss	both	14669916-16831960
V-021	16p13.11	copy gain	both	16200143-16292148
V-7015	16p13.2	copy loss	autism	6764193-7072088
V-064	16p12.2	copy loss	both	21328596-21976691
V-063	16p11.13	copy loss	both	14669916-16831960
V-7009	16p11.2	copy gain	autism	28949609-30271188
V-7048	16p11.2	copy loss	autism	28473346-28949550
V-7082	16p11.2	copy loss	autism	28012439-30290867
V-7096	16p11.2	copy gain	autism	28199143-29678569
L-AE-28	17p12	copy loss	both	14014324-15492482
L-AE-02	17p11.2	duplication	both	16755641-20147193
V-7097	18p11.31-11.23	copy gain	autism	7050712-7859424
V-4001	18q22.1	copy gain	both	63799497-64741418
V-7032	19p13.3	copy gain	autism	781639-810851
V-7064	19q12	copy gain	autism	19857596-20596412
V-5003	19q13.32-13.33	copy gain	both	53085957-55852469
V-7098	21q22.11-22.12	copy gain	autism	34657967-34832077
V-080	21q23.3	copy gain	both	43904642-44267159
V-032	22q11.21	copy gain	both	18919048-21939923
V-7080	22q13.1	copy loss	autism	38092709-38106149
V-7062	22q13.31-13.33	copy loss	autism	45145508-49691432
L-AA-07	Xp22.33-q28	triple X	autism	216519-154881514
L-AE-02	Xp22.33	copy loss	both	3761569-3863478
L-AA-06	Xp22.32	copy gain	autism	8598757-8701466
V-092	Xp22.31	copy gain	both	6279319-8199482
V-4001	Xp22.31	copy gain	both	6289319-7764729
V-7080	Xp22.2	copy loss	autism	16775568-16804240
V-7007	Xp21.1	copy loss	autism	32818043-33628679
V-021	Xp12	copy gain	autism	65718497-65953240
